# Update of the EMHG database on genetic variants in type 1 ryanodine receptor and their possible impact on phenotype

**DOI:** 10.1186/1471-2253-14-S1-A12

**Published:** 2014-08-18

**Authors:** Florian Grütter, Thierry Girard

**Affiliations:** 1 University of Basel, Basel, 4003, Switzerland; 2 Department of Anesthesia, University Hospital of Basel, Basel, 4031, Switzerland

## Background

The type 1 ryanodine receptor (RYR1) is expressed in human skeletal muscle and plays a key role in calcium homeostasis. Most patients with malignant hyperthermia (MH) have mutations in the RYR1 gene.

Today, the gold standard for MH diagnosis is an invasive open muscle biopsy followed by the in-vitro-contracture-test (IVCT). Less invasive diagnosis using molecular genetic methods is of increasing importance, but knowledge about genetic variants in RYR1 is limited. The European MH Group (EMHG) RYR1 mutation database is not up-to-date and lacks information on a significant number of more recently identified variants or further knowledge on variants already in the database. Therefore, the need for an update in order to expand the use of molecular genetic testing is required.

The aim of this study was to collect information about variants in the RYR1 gene and their functional effect. Finally this information will be imported into the EMHG RYR1 mutation database.

## Materials and methods

PubMed was used for data collection. For the search, the three keywords "Malignant Hyperthermia", "Mutation" and "Ryanodine Receptor Calcium Release Channel" were used; the operator was "AND". Publication dates from the beginning of 2006 to the end of 2012 were selected. However, some older and newer studies were also included if they seemed important for the gain of information. Additionally, the considered papers had to be relevant for the human species. The software EndNote X7 (Thomson Reuters, Carlsbad, CA, USA) was used for administration of references and data was collected and processed in Microsoft Excel for Mac 2011 (Microsoft Corporation, Redmont, WA, USA).

## Results

Altogether 62 publications were found according to the search algorithm. In the newly created database, an overall of 316 variants were gathered. 143 variants were not yet in the EMHG database, 129 of them not functionally characterized or non-pathogenic, 13 functionally characterized and putatively causative and one causative (p.H4833Y), respectively.

**Figure 1:**
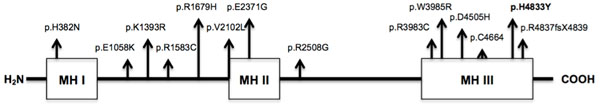
Location of the newly Identified and functionally characterized RYR1 variants in the RYR1 gene. MH I-III: Hot spots in which most MH associated mutations are found today. MH I = N-terminal region (p.C35-p.R614), MH II = central region (p.D2129-p.R2458), MH III = C-terminal region (p.l3916-p.G4942). The variant p.H4833Y (in bold) fulfils all criteria to be used in genetic testing and is therefore a causative mutation.

Among another 11 variants which were already in the EMHG database two (p.R530H, p.R2336H) can be classified as causative (Figure 1).

## Conclusions

This study shows that important additional information on variants in RYR1 was published since 2006. However, only three mutations (p.R530H, p.R2336H and p.H4833Y) can be newly classified as causative mutations according to the EMHG guidelines. The main reason for this disappointing conclusion is the fact that the criterion "co-segregation with the disease in at least two pedigrees" according to the EMHG guidelines was missing in otherwise MH causative mutations. Nevertheless, this update of the database gives easier access to genetic information and emphasizes the increasing significance of genetic testing.

